# A SNP in intron 1 of TSHR controls its thymic expression and susceptibility to Graves’ disease suggesting central tolerance failure in pathogenesis

**DOI:** 10.1186/1479-5876-9-S2-P7

**Published:** 2011-11-23

**Authors:** Maria del Pilar Armengol, Rosa Faner, Lars-Olivier Tykocinski, Anna Lucas-Martin, Marta Ruiz-Riol, Manel Juan, Bruno Kyewski, Ricardo Pujol-Borrell, Roger Colobran

**Affiliations:** 1 Genomics, Institut d’Investigació en Ciències de la Salut Germans Trias i Pujol, Badalona, Spain; 2 Developmental Immunology - Tumour Immunology Program, Cancer Research Center, Heidelberg, Germany; 3 Immunology, Valle Hebron Research Institute - Universitat Autonoma de Barcelona, Barcelona, Spain

## 

Graves’ disease (GD) is the paradigm of an anti-receptor autoimmune disease with agonistic auto-antibodies against the thyrotropin receptor (TSHR) being the underlying pathogenic mechanism. TSHR belongs to the category of tissue-restricted antigens (TRAs), which are expressed by medullary thymic epithelial cells (mTECs) and thereby induce central T cell tolerance. In order to understand the association between TSHR gene polymorphisms and GD we tested the hypothesis that TSHR gene variants affect susceptibility to GD by influencing levels of TSHR transcription in the thymus. The results indicate that thymic glands from normal children homozygous for the rs179247 predisposing allele of TSHR had significantly fewer TSHR mRNA transcripts than carriers of the protective allele. In addition, in heterozygous, the TSHR predisposing allele was expressed at a lower level than the protective one as demonstrated by Allele Specific Transcript Quantification. The effect of TSHR SNP rs179247 was thymus-specific and not observed in thyroid glands. These results constitute first evidence for the involvement of central tolerance in the loss of tolerance to TSHR in GD and underscore the concept that variable expression levels of major target autoantigens in the thymus influence the predisposition to autoimmunity presumably by changing the threshold of tolerance.

